# Anti-DNA-IgM Favors the Detection of NET-Associated Extracellular DNA

**DOI:** 10.3390/ijms24044101

**Published:** 2023-02-17

**Authors:** Han Wang, Antonia Margarethe Stehr, Jeeshan Singh, Leticija Zlatar, Arndt Hartmann, Katja Evert, Elisabeth Naschberger, Saskia von Stillfried, Peter Boor, Luis E. Muñoz, Jasmin Knopf, Michael Stürzl, Martin Herrmann

**Affiliations:** 1 Department of Internal Medicine 3, Rheumatology and Immunology, Friedrich-Alexander-Universität Erlangen-Nürnberg (FAU), Universitätsklinikum Erlangen, 91054 Erlangen, Germany; 2 Deutsches Zentrum für Immuntherapie (DZI), Friedrich-Alexander-Universität Erlangen-Nürnberg (FAU), Universitätsklinikum Erlangen, 91054 Erlangen, Germany; 3 Division of Molecular and Experimental Surgery, Friedrich-Alexander Universtität Erlangen-Nürnberg (FAU), Universitätsklinikum Erlangen, 91054 Erlangen, Germany; 4 Comprehensive Cancer Center Erlangen-EMN, Universitätsklinikum Erlangen, 91054 Erlangen, Germany; 5 Institut für Pathologie, Friedrich-Alexander-Universität Erlangen-Nürnberg (FAU), Universitätsklinikum Erlangen, 91054 Erlangen, Germany; 6 Institut für Pathologie, Universität Regensburg, 93053 Regensburg, Germany; 7 Institute of Pathology, University Clinic of the RWTH Aachen, 52074 Aachen, Germany

**Keywords:** extracellular DNA, NETs, anti-DNA-IgM

## Abstract

During inflammatory responses, neutrophils enter the sites of attack where they execute various defense mechanisms. They (I) phagocytose microorganisms, (II) degranulate to release cytokines, (III) recruit various immune cells by cell-type specific chemokines, (IV) secrete anti-microbials including lactoferrin, lysozyme, defensins and reactive oxygen species, and (V) release DNA as neutrophil extracellular traps (NETs). The latter originates from mitochondria as well as from decondensed nuclei. This is easily detected in cultured cells by staining of DNA with specific dyes. However, in tissues sections the very high fluorescence signals emitted from the condensed nuclear DNA hamper the detection of the widespread, extranuclear DNA of the NETs. In contrast, when we employ anti-DNA-IgM antibodies, they are unable to penetrate deep into the tightly packed DNA of the nucleus, and we observe a robust signal for the extended DNA patches of the NETs. To validate anti-DNA-IgM, we additionally stained the sections for the NET-markers histone H2B, myeloperoxidase, citrullinated histone H3, and neutrophil elastase. Altogether, we have described a fast one-step procedure for the detection of NETs in tissue sections, which provides new perspectives to characterize neutrophil-associated immune reactions in disease.

## 1. Introduction

Deoxyribonucleic acid (DNA) is a polymer made up of two polynucleotide chains coiling around each other to form a double helix. In the nucleus, the DNA is responsible for carrying genetic instructions for gene regulation and heredity [1]. Upon cell death, stress or injury, DNA may leak to extranuclear and extracellular spaces. This shifts its primary function from storing genetic information to driving inflammation and thrombosis [2]. In the process of DNA leakage, chromatin decondenses, and the extracellular charged phosphodiester backbone and its expanded polymeric structure provide habitats for intermolecular interactions [3]. For instance, it may gain antigenic activity and bind to serum components including autoantibodies of patients with Systemic lupus erythematosus (SLE); this may generate massive amounts of pathogenic immune complexes [4,5].

Cell death is the primary source of extracellular DNA (ecDNA). This DNA is considered a byproduct of cell death and, generally, it will be eliminated swiftly without activation of the host’s immune system [6,7,8]. However, if the immune function is compromised or if processing capacity reaches saturation, ecDNA can be dangerous, as it interacts with various nucleic acid sensors. The latter are part of the internal host defense and are triggered by DNA from bacteria or viruses as well as by extranuclear self-DNA produced by cellular stress or enriched by impaired nuclease activity [9,10].

In addition to unintended and programmed cell death, neutrophil extracellular traps (NET) formation is another source of ecDNA [11]. NET formation is an intricate program of polymorphonuclear granulocytes (PMNs) that involves the movement of DNA from the nucleus to the cytoplasm. In the presence of peptidyl arginine deiminase 4 (PAD4) or neutrophil elastase (NE), histones are citrullinated or cleaved, respectively. This facilitates the externalization of chromatin [12,13,14]. Dynamics in cortical actin polymerization and depolymerization indicate an additional phase in the transfer of nuclear DNA via the cytoplasm to the extracellular space [15]. This process is regulated by NE via the binding and degradation of F-actin [16]. In the extracellular context, the decondensed DNA as a backbone of NETs is decorated with assorted proteins [17], including histones, proteases, and glycosidases.

Since the discovery of NET formation, many attempts have been made to develop methods for the accurate and stable detection of NETs in vitro and in vivo. Fluid samples, such as cell culture supernatants, serum or cerebrospinal fluid have been analyzed using ELISA or flow cytometry to quantify the NETs [18,19], which, although boosting the measurement’s objectivity, sacrifices spatiality and intuitiveness. For now, staining with small DNA-specific fluorochromes and immunofluorescence for NET-borne proteins still remains the most sensitive and versatile method for the detection of NETs in cell culture and in tissues.

There are numerous NETs-related markers, such as NE, myeloperoxidase (MPO) and (citrullinated) histones. Researchers typically select one or more to co-localize with DNA dyes such as DAPI or SYTOX™ Green in order to detect NETs [20]. This can be a significant disadvantage because small molecular dyes such as DAPI can diffuse into the nuclei. There they emit an extremely strong fluorescence signal that outshines the weak signals representing the spread of decondensed ecDNA, which has a much lower local DNA concentration.

We found that, in the case of decondensed and extranuclear DNA, detection is significantly enhanced when using immunofluorescence with an immunoglobulin M (IgM) antibody against DNA. We validated this approach using biopsies from coronavirus disease 2019 (COVID-19) patients’ skin, liver, kidney, placentas, and lungs as well as colon tumor biopsies stained with anti-DNA-IgM, anti-histone 2B (H2B)-IgG, anti-MPO-IgG, anti-citrullinated histone H3 (citH3)-IgG and anti-NE-IgG antibodies. Additionally, we stained NETs formed upon stimulation of freshly isolated neutrophils with anti-DNA-IgM and the NETs marker mentioned above. DAPI or SYTOX™ Green served as nuclear counterstains.

## 2. Results

### 2.1. Anti-DNA-IgM Is Superior in Detecting Decondensed DNA In Vivo

**Figure 1:**
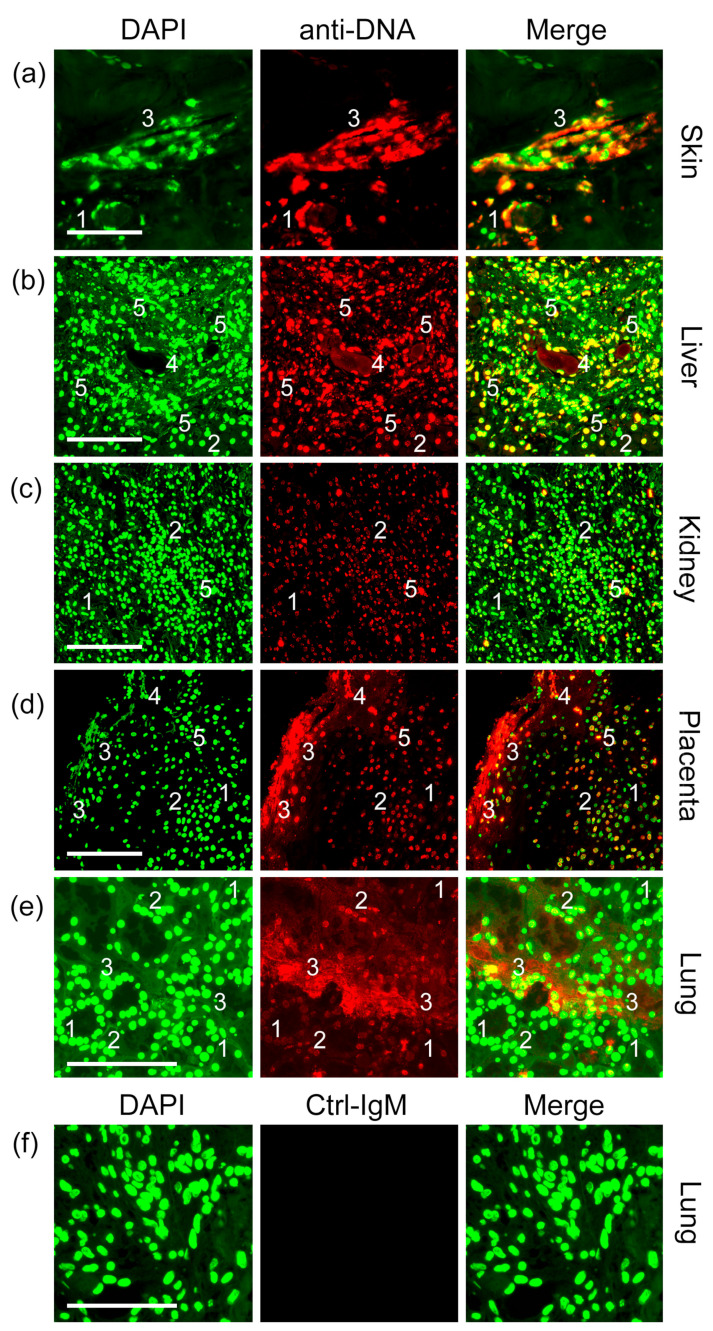
Anti-DNA-IgM antibody surpasses DAPI in staining decondensed DNA. We analyzed paraffin sections from specimens of (**a**) skin (n = 7), (**b**) liver (n = 7), (**c**) kidney (n = 7), (**d**) placenta (n = 8), and (**e**) lung (n = 7) of patients with COVID-19. DAPI (green), anti-DNA-IgM (red), and merged images are shown in the left, middle, and right panels, respectively. (**f**) Isotype control. Note, condensed nuclei show a strong DAPI signal and (1) no or (2) faint anti-DNA-IgM staining. (3) The spread extracellular DNA (ecDNA) of neutrophil extracellular traps (NETs) is preferentially stained using the anti-DNA-IgM antibody and (4) some vessels display intravascular DNA which is below the threshold of detection using DAPI. (5) Nuclear remnants in cellular debris are equally stained by DAPI and anti-DNA-IgM. Scale bar = 100 µm.

The presence of ecDNA can be observed in various organs of COVID-19 patients, such as skin, liver, kidney, placenta and lung. The staining pattern of ecDNA is distinct from that of normal nuclei, showing irregular shapes such as filaments and patches (Figure 1 and Figure S1). The dense DNA of the nuclei has a large amount of DAPI bound per unit space. The ecDNA has a more diffuse chromatin structure than the dense nuclear DNA, which leads to a lesser amount of DAPI bound per unit space. This decrease in DAPI binding leads to a weaker fluorescence intensity, even below the detectable limit (Figure 1b,d). Meanwhile, the intensive fluorescence signals, due to high amounts of bound DAPI, very easily obscures the detection of ecDNA, creating a blind zone in our field of view (Figure 1a,d,e).

**Figure 2:**
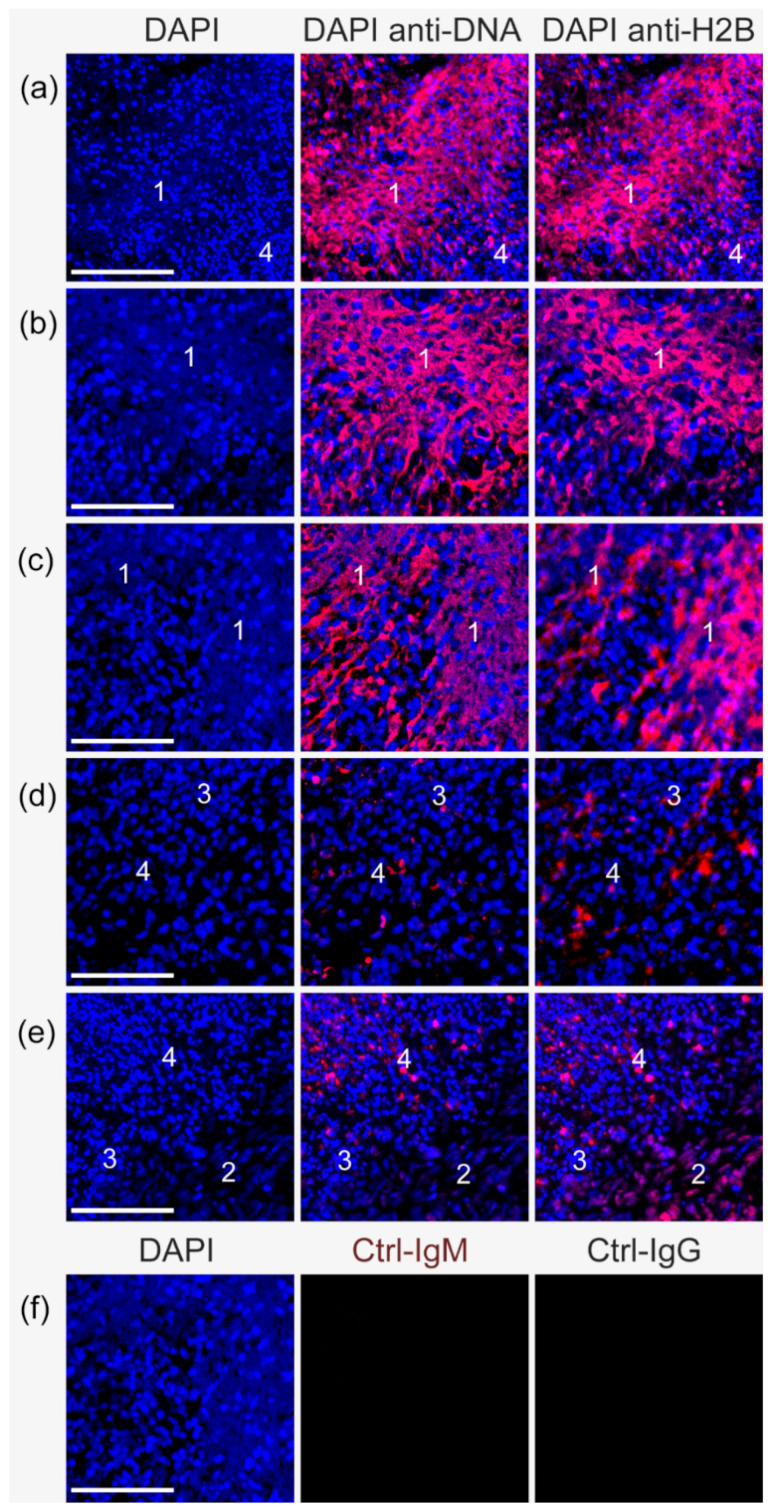
Anti-DNA-IgM antibody staining favors the detection of ecDNA in DNA rich and poor tissue specimens. Epifluorescence and confocal images from colon tumor patients. Stains: DNA by DAPI (blue) and chromatin by anti-DNA-IgM-TRITC and anti-H2B-IgG-Cy5 (both displayed in red). (**a**–**c**) extracellular DNA-rich and (**d**,**e**) extracellular DNA-poor tissue sections are displayed. (1) Anti-DNA-IgM presents an intensive fluorescence signal in the extracellular environment that co-localized with anti-H2B-IgG. (2) Anti-H2B-IgG also stains intranuclear decondensed chromatin. The co-localization of anti-H2B and anti-DNA-IgM confirms its nature as NETs-borne decondensed chromatin. Cells with condensed DNA in their nuclei show a high DAPI signal and (3) no or (4) faint anti-DNA-IgM and anti-H2B-IgG signal. (**f**) isotype control staining. Scale bar = 100 µm.

Anti-DNA-IgM antibodies bind better to extranuclear chromatin and NETs-related DNA, forming an extensive reticulation, and increase their visibility in fluorescence stainings of tissue (Figure 2). This is true for tissue specimens rich in ecDNA (Figure 2a–c) but also for locations with low amounts of ecDNA (Figure 2d,e). In contrast, faint lamellar DNA structures can also be observed using DAPI staining but with the drawback of overexposure and even visual distortion of DNA in the nucleus. The anti-DNA-IgM signal co-localizes with anti-H2B-IgG (Figure 2a–c), a marker for relaxed decondensed chromatin [20] (Figure 2e), whereas the nuclei of intact cells are robustly stained by DAPI with no–or only faint–signals using anti-DNA-IgM or anti-H2B (Figure 2a,d,e).

**Figure 3:**
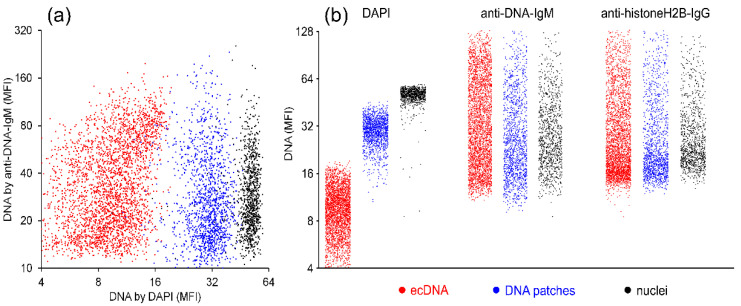
Morphometry revealed the advantage of anti-DNA-IgM for the detection of decondensed DNA. (**a**) DNA is indicated using the mean fluorescence intensity (MFI) of DAPI and anti-DNA as three classifications—ecDNA (red), DNA patches (blue), and nuclear (condensed) DNA (black). (**b**) Distribution of three forms of DNA with DAPI, anti-DNA-IgM, and anti-H2B-IgG. When compared to anti-DNA-IgM, DAPI displays a high fluorescence for chromatin from condensed nuclei. In addition, anti-DNA-IgM exhibits an analogous fluorescence profile of ecDNA with anti-H2B.

Analyses by morphometry from chromatin of colon tumor biopsies (Figure 2) revealed differences in the mean fluorescence intensity (MFI) signal of the DAPI vs. anti-DNA-IgM signal (Figure 3). These differences in the MFI could be classified into three categories of DNA/chromatin: (1) ecDNA (red), (2) DNA patches (blue), and (3) nuclear (condensed) DNA (black). Nuclear (condensed) DNA showed the highest MFI in DAPI staining, DNA patches resulted in reduced signals and ecDNA showed the lowest MFI (Figure 3a). When comparing the different groups of DNA/chromatin, it was evident that the highest MFI signal for DAPI was detected within the nuclear DNA and the lowest in ecDNA, but for anti-DNA-IgM and anti-histone H2B, the MFI was similar in all three groups of DNA/chromatin (Figure 3b).

**Figure 4:**
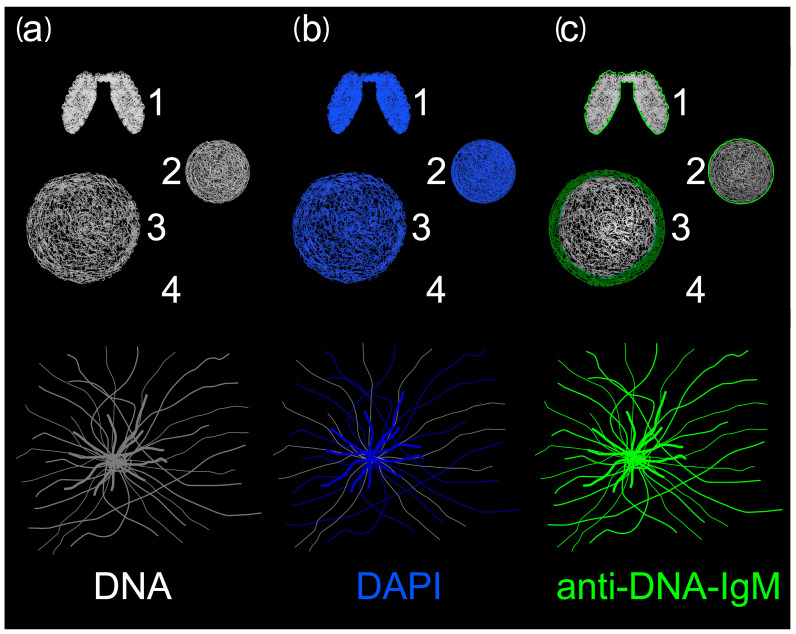
Schematic display of the immunofluorescence signal derived from DAPI and anti-DNA-IgM staining. (**a**) DNA is shown in grey. (**b**) DAPI, and (**c**) anti-DNA-IgM are shown in blue and green, respectively, upon staining of DNA. (1) Condensed mitotic DNA exhibits high DAPI and faint anti-DNA-IgM signal mostly at the nucleoplasmic borders of the nucleus. (2,3) Extranuclear DNA relaxes, increases anti-DNA-IgM binding and lowers the number of DAPI bindings sites with a subsequent decrease in DAPI-MFI. (4) Extracellular DNA is decondensed and therefore well accessible for the anti-DNA-IgM antibody. Some parts of the decondensed DNA of NETs are below the detection limit by DAPI but can be visualized upon staining with anti-DNA-IgM.

During DNA decondensation, the relaxation of chromatin leads to the accessibility of epitopes for the binding of anti-DNA-IgM antibodies. This results in a more intense fluorescence signal. Conversely, the signal intensity of DNA intercalating dyes such as DAPI decreased due to chromatin decondensation and a subsequent decrease in the DNA density (Figure 4).

### 2.2. Anti-DNA-IgM Is Suitable for High Sensitive Detection of NETs and ecDNA

**Figure 5:**
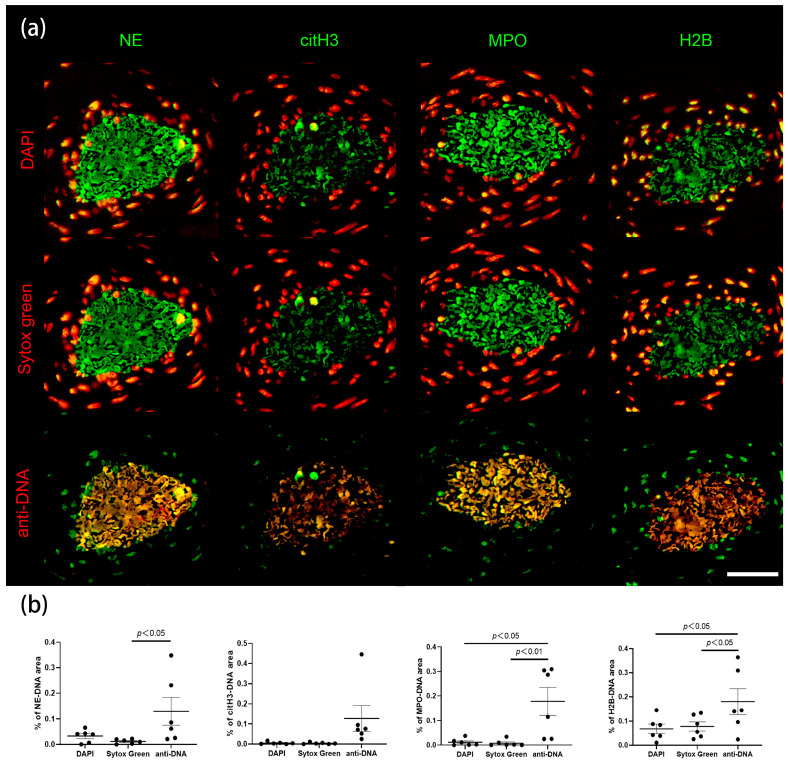
Immunofluorescence staining and quantification of NETs in tissues from COVID and colon tumor patients. (**a**) DNA is stained using DAPI (red, first row), SYTOX™ Green (red, second row) and anti-DNA-IgM (red, last row). NETs-associated proteins are stained using anti-NE-IgG (green, first column), anti-citH3-IgG (green, second column), anti-MPO-IgG (green, third column) and anti-H2B-IgG (green, last column). Scale bar = 50 µm. (**b**) Measurement of co-localization area ratio of tissue sections in six spots, N = 2. Data were presented as mean ± SEM. *p* < 0.05 is significant.

To test the specificity of this observation, we utilized the brighter DNA dye SYTOX™ Green next to DAPI to compare with anti-DNA-IgM. We employed four NETs markers: NE, citH3, MPO and H2B co-stained with the three DNA dyes. Tissue sections of placenta from COVID patients revealed a high co-localization of anti-DNA-IgM with other NETs markers compared with DAPI and SYTOX™ Green (Figure 5 and Figure S2). Simultaneously, anti-DNA-IgM expression was reduced compared to anti-H2B-IgG in extracellular chromatin (Figure 5a). This indicates that intact nuclei can be clearly identified using small DNA stains, but are hardly penetrated by anti-DNA-IgM. (Figure 5a). Although DAPI and SYTOX™ Green can stain ecDNA to some extent, they are likely to be obscured by the high signal of nuclear DNA (Figure S2). Anti-H2B-IgG not only stains the extracellular chromatin but also intracellular chromatin, while anti-DNA-IgM tends to bind more to DNA in the extracellular space (Figure 5a). We also performed quantification of the area ratio of co-localization between the DNA stains and neutrophil proteins, which indicates NETs. (Figure 5b). This quantification clearly shows that anti-DNA-IgM stains more NETs relative to DAPI and SYTOX™ Green in the same spot, which confirms the high sensitivity of anti-DNA-IgM in the detection of NETs. (Figure 5b).

In vitro induction of NETs with various stimuli has become a preferred method for the analysis of neutrophils and NETs from healthy donors and patients due to its robust-ness [21]. Hence, we validated the use of anti-DNA-IgM for the in vitro detection of NETs.

**Figure 6:**
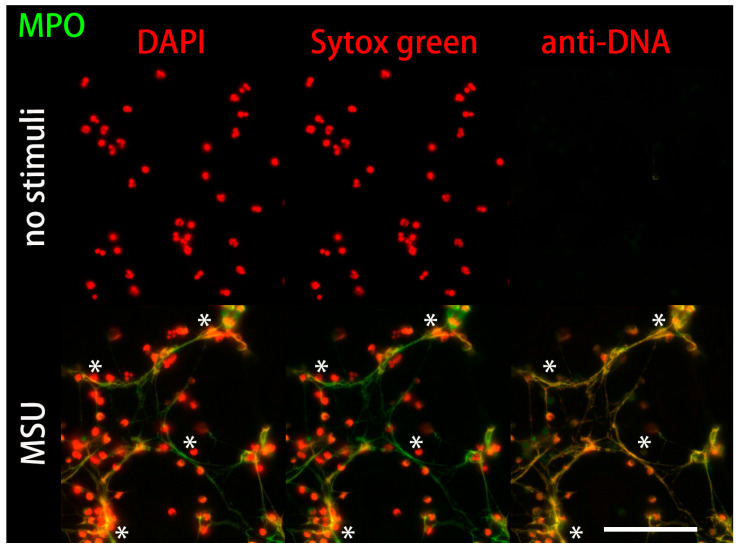
Anti-DNA-IgM stains DNA from NETs with high sensitivity in vitro. Freshly isolated neutrophils from healthy donors were cultured without stimulation (no stimuli), or stimulated with monosodium urate crystals (MSU) to trigger NET formation. Then the samples were stained with the small DNA dyes DAPI (red, first column), SYTOX™ Green (red, second column) or anti-DNA-IgM (red, last column). Fibrous decondensed DNA of NETs was detected robustly by anti-DNA-IgM but virtually not by DAPI and SYTOX™ Green. It co-localized with MPO (asterisk). Unstimulated viable cells show no anti-DNA-IgM signal, but display strong DAPI and SYTOX™ Green signals. Scale bar = 100 µm.

Unstimulated neutrophils retain a lobulated nucleus characterized by a high intensity of the DAPI and SYTOX™ Green signal. In contrast, anti-DNA-IgM did not penetrate into the nuclei. After treatment with stimuli such as monosodium urate crystals (MSU), the nuclei of the neutrophils gradually decondensed and their chromatin was released into the extracellular space. This chromatin was additionally decorated with NET-associated proteins such as MPO. Co-localization of anti-DNA-IgM and MPO exhibited the morphology of fine fibers of NETs, which can hardly be detected using the small DNA stains DAPI and SYTOX™ Green (Figure 6).

## 3. Discussion

Various cellular barriers segregate the nucleus from the extracellular environment. For instance, the plasma membrane, a dynamic, lipophilic structure, can impede the flow of large, hydrophilic, or charged molecules [22]. The large and negatively charged DNA cannot cross this barrier. The nuclear pore complex (NPC) of the nuclear membrane serves as a further barrier to diffusive molecules of varying sizes. Molecules larger than 45 kDa cannot penetrate the nuclei unless they possess a special targeting signal referred to as nuclear localization sequence (NLS) [23]. DNA fragments smaller than 250 base pairs can freely diffuse into the nucleus, whereas bigger fragments cannot [24,25,26]. SYTOX™ Green is a commonly used bright fluorescent dye that binds specifically to double-stranded DNA (dsDNA). DAPI is a minor groove fluorescent probe with a molecular weight of 350 Da. It may enter the nucleus via NPC via passive diffusion and bind to the AT-rich region of double-stranded DNA [27]. At this stage, there is a substantial increase in fluorescence emission (20–30 times higher than in the unbound state) [28]. Conversely, when DAPI attaches to single-stranded DNA, only modest alterations in the fluorescence emission can be seen [29,30]. In certain circumstances, DAPI may stain protein aggregates, blurring the staining of the nucleus [31].

Regardless of how cell death has been induced, ecDNA has a main size of roughly 160 bases—the size of a single nucleosome [32,33]. This size is identical to that seen in studies of circulating DNA [34]. IgM molecules bind with low affinity to small DNA fragments in vitro through electrostatic interactions between negatively charged DNA phosphates and positively charged protein amino groups, with further stabilization by hydrogen bonding and metal affinity [35]. Our results obtained with the high affinity anti-DNA-IgM show that the immunodetection of ecDNA is superior to DAPI or SYTOX™ Green [36,37]. Given that IgM has a molecular weight of >900 kDa, an overall diameter of 30 nm, and a core diameter of 16 nm [38], it displays a very low diffusion velocity to enter compacted viable nuclei. Fully condensed chromatin from unstimulated neutrophils gives virtually no anti-DNA-IgM signal. At the same time, anti-DNA-IgM faintly stains sectioned nuclei due to the highly condensed state of the latter [39]. In our study, we have also investigated the impact of permeabilization during the staining procedure on the staining pattern of anti-DNA-IgM in tissues and cells. We found that permeabilization of tissues or cells had a minimal effect on the staining results (Figures S3–S5). This is likely because the tissue sections were relatively thin at 4 µm, compared to the size of human cells, which are typically 10–100 µm in diameter. This suggests that some, but not all, cell membranes had already been damaged by sectioning before the tissue was stained.

Identifying dysregulated development and clearance of NETs in infections, sepsis, autoimmune diseases, thrombosis, metabolic disorders, and cancer is critical for understanding the nature of these diseases. The DNA that builds the backbone of NETs is formed by certain mechanisms. Nuclear envelope rupture, nuclear chromatin depolymerization, and plasma membrane rupture are essential biological events for the extracellular release of nuclear chromatin in suicidal NET formation. In contrast to suicidal NET formation, vital NET formation has been described as the extracellular release of mitochondrial or nuclear DNA without the loss of plasma membrane integrity [40,41]. There have been reports on the use of anti-DNA antibodies directed against extracellular chromatin regardless of whether the DNA is of nuclear or mitochondrial origin [41,42]. It has a capacity to differentiate between condensed and compacted chromatin within neutrophils of viable appearance and depolymerized DNA ejected from former neutrophils that exhibit now characteristics of NETs. Although there have been some markers to label NETs, they usually identify NETs using the co-localization of NETs-bound proteins and DNA, stained with small fluorescent molecules. H2B, as one of the NETs markers, not only stains released chromatin as present in NETs and netting neutrophils but also compact the chromatin of a normal nuclei. A major disadvantage is that the low signals for ecDNA are outshone by the strong signals from intact nuclei. This often leads to false negative results for the detection of ecDNA and NETs. The use of immunodetection of ecDNA and NET-borne proteins will improve the analysis of NETs in tissue sections.

## 4. Materials and Methods

### 4.1. Ethical Approval

All patients included in this investigation provided written and informed consent. All experiments on human material were performed according to the 1964 Helsinki Declaration and its later amendments or comparable ethical standards for research involving human subjects. In addition, institutional ethical approval was obtained from each local Ethical Committee (permit #193_13B; permit #174_20B; permit #159_15B, EK 092/20; EK 119/20; EK 460/20).

### 4.2. Tissue Collection

Tissue specimens of kidney (n = 7), liver (n = 7), lung (n = 7), skin (n = 7) and placenta (n = 8) from patients with COVID-19 and colon tumors (n = 85) were anonymously obtained from the pathology departments in Erlangen, Regensburg and Aachen.

### 4.3. Isolation of Neutrophils

Neutrophils were isolated from freshly drawn peripheral blood of healthy donors (NHD). The blood was drawn from a venipuncture into K3 EDTA (Sarstedt AG & Co. KG, Nümbrecht, Germany) tubes and diluted with phosphate buffered saline (PBS, Thermo Fisher Scientific, Waltham, MA, USA) to a final volume of 35 mL in a 50 mL tube (Greiner bio-one GmbH, Kremsmünster, Austria). The mixture was carefully pipetted on the top of 15 mL Ficoll-Diatrizoate density gradient solution (Lymphoflot, Bio-Rad, Hercules, CA, USA) and centrifuged at 1400 rpm for 30 min at room temperature. PMNs were collected and treated for two brief cycles of hypotonic lysis with ice-cold 36 mL deionized water for 20 s. Osmolarity was restored by addition of 4 mL 10× PBS. Isolated PMNs were resuspended in PBS without calcium and magnesium for counting using the LUNA-FL™ Dual Fluorescence Cell Counter (Logos biosystems, Anyang, Gyeonggi-do, Republic of Korea). The PMN were adjusted to a concentration of 2 million/mL in RPMI-1640 without phenolred (Thermo Fisher Scientific) substituted with 2 mM L-glutamine (Thermo Fisher Scientific) and Penicillin/Streptomycin (Thermo Fisher Scientific) and utilized immediately for the in vitro assays.

### 4.4. Staining of Tissue Sections

Paraffinized tissue specimens were deparaffinized for 2 h at 60˚C. Then, traces of any residual paraffin were removed by immersing the tissue specimens in xylol twice for 15 min each. Next, the tissue sections were rehydrated in decreasing concentrations of alcohol (100%–96%–85%–70%) for 2 min, twice at each concentration. The epitopes were retrieved in a target retrieval solution pH 9 (TRS) (Agilent, Santa Clara, CA, USA) for 2 h at 70 °C for neutrophil elastase (NE), citrullinated Histone H3 (citH3), myeloperoxidase (MPO), and Histone H2B antibody staining or for 20 min at 90 °C using a target retrieval solution pH 6 (Agilent) for DNA antibody staining for the colon tumor biopsies. The epitopes of biopsies from different organs of patients with COVID-19 were all retrieved using 10 mM sodium citrate buffer pH 6 (Merck KGaA, Darmstadt, Germany) for 20 min at 90 °C. The tissue sections were cooled and brought to room temperature after epitope retrieval. Then the sections were either permeabilized with 0.5% Triton in 1× TBS for 6 min and followed by blocking for 1 h in the blocking buffer or directly blocked for 1 h in the blocking buffer. The blocking buffer solution contained 10% normal donkey serum (Dianova GmbH, Hamburg Germany), or 10% Fetal Calf Serum (FCS), 2% bovine serum albumin (BSA) (Sigma-Aldrich, Saint Louis, MO, USA) in 1× TBS at room temperature. The colon tumor tissue sections were then incubated overnight at 4 °C with the following primary antibodies: chicken anti-human H2B antibody (1:1000, ab134211, Abcam, Cambridge, UK, polyclonal IgY), mouse anti-human DNA antibody (1:100, #CBL186, Merck KGaA, monoclonal IgM), rabbit anti-human NE antibody (1:100, ab68672, Abcam, Cambridge, UK, polyclonal IgG), rabbit anti-human citH3 antibody (1:300, ab5103 Abcam, Cambridge, UK, polyclonal IgG), or rabbit anti-human MPO antibody (1:100, ab9535, Abcam, Cambridge, UK, polyclonal IgG). Isotype control stainings were performed on consecutive tissue specimens. For this, the sections were incubated with chicken IgY isotype (1:100, ab50579, Abcam), rabbit IgG isotype (1:1000, AB105-C, R&D Systems, Minneapolis, MN, USA) and mouse IgM isotype control (1:2000, 02-6800, Thermo Fisher Scientific) and blocking buffer overnight at 4 °C. For tissue sections from various organs of patients with COVID-19, rabbit anti-human NE antibody (1:100, ab68672, Abcam, polyclonal IgG), mouse anti-histone H2B antibody (1:100, ab52484, Abcam, monoclonal IgG), rabbit anti-human citH3 antibody (1:300, ab5103 Abcam, Cambridge, UK, polyclonal IgG) and rabbit anti-human MPO antibody (1:100, ab9535, Abcam, Cambridge, UK, polyclonal IgG) were used as primary antibodies. Following the overnight incubation at +4 °C, the following secondary antibodies were applied for the colon tumor tissue sections: Cy™2 AffiniPure donkey anti-rabbit IgG (H+L) (1:400, 711–225-152, Jackson ImmunoResearch Europe Ltd., Ely, UK), Cy™3 AffiniPure donkey anti-chicken IgY (H+L) (1:400, 703–165-155, Jackson ImmunoResearch Europe Ltd.); Alexa Fluor™ 647 goat anti-mouse IgM (Heavy chain) (1:100, A21238, Thermo Fisher Scientific) for 1 h at room temperature in the dark. For the tissue sections from COVID-19 patients, the following secondary antibodies were applied: Alexa Fluor™ 568 goat anti-rabbit IgG (H+L) (1:400, ab175471, Abcam, Cambridge, UK, polyclonal IgG), Cy™5 AffiniPure goat anti-mouse IgM (H+L) (1:400, 115-175-075, Jackson ImmunoResearch Europe Ltd.) and Alexa Fluor™ 555 goat anti-mouse IgG (H+L) (1:400, ab150114, Abcam, Cambridge, UK, polyclonal IgG). The sections were then counterstained for nuclear staining with DAPI (ThermoFisher) and Sytox^TM^ Green (ThermoFisher) mounted with DAKO fluorescence aqueous mounting medium (S3023, Agilent).

### 4.5. NET Formation In Vitro

100 µL/well of freshly isolated PMN in RPMI-1640 medium at a concentration of 2 million/mL were seeded in Nunc™ Lab-Tek™ Permanox chamberslides (Thermo Fisher Scientific). The chamber slides were incubated with 50 pg/cell monosodium urate (MSU) crystals for 4 h at 37 °C to allow NET formation. The medium alone served as unstimulated control. Afterwards, samples were fixed using 2% paraformaldehyde for 20 min at RT. After washing of the samples three times with PBS, NETs/PMN were permeabilized using 0.1% Triton X-100 in PBS for 5 min at RT before blocking with 10% FCS, 2% BSA in PBS for 1 h at RT. Mouse anti-DNA-IgM (CBL186, Merck KGaA; diluted 1:100) and rabbit anti-human MPO antibody (1:100, ab9535, Abcam, Cambridge, UK, poly-clonal IgG) were incubated in blocking buffer ON at +4 °C. After washing three times with PBS, Alexa Fluor™ 568 goat anti-rabbit IgG (H+L) (1:400, ab175471, Abcam, Cambridge, UK, polyclonal IgG) and Cy™5 AffiniPure goat anti-mouse IgM (H+L) (1:400, 115-175-075, Jackson ImmunoResearch Europe Ltd.) in blocking buffer were used as a secondary antibody. The secondary antibodies were incubated with the DNA stains DAPI (ThermoFisher) and Sytox^TM^ Green (ThermoFisher) for 1 h at RT. After another washing step with two times PBS and one-time deionized water, the chambers were removed and slides were mounted using DAKO fluorescent mounting medium (Agilent).

### 4.6. Microscopy

Immunofluorescence photos of COVID-19 specimens were taken using an Aperio VERSA 8 Slide Scanner (Leica Biosystems, Wetzlar, Germany) and analyzed using the Aperio ImageScope Software v12.4.3.7001 (Leica Biosystems). Colon tumor tissues were imaged using a Leica DM6000 B epifluorescence microscope (Leica Biosystems) or a Leica TCS SP8 confocal microscope (Leica Biosystems).

Fluorescence microscopy of in vitro NETs was performed with a BZ-X700 microscope (Keyence Corporation, Osaka, Japan). To optimize the depth of field, quick full focus of the area of interest in the BZ-X Viewer Software version 01.03.00.05 (Keyence Corporation) was performed.

### 4.7. Morphometry

We conducted morphometry with Adobe Photoshop CC Version 19.1.8 (Adobe Inc, Mountain View, CA, USA). RGB-channels were segmented into DAPI, DNA and H2B with Photoshop. Contrast ratio of each part were kept constant over the whole series in an analysis after calibrating on isotypes to avoid artifacts. The parameters used for particle segmentation were set automatically by scrolling through and checking the image stacks. The segmented object outlines were superimposed on RGB-merged stacks of the DAPI, DNA, and H2B. The result of particle analysis was imported into Microsoft Excel 2016 (Microsoft Corporation, Redmond, WA, USA) and represented in combined line diagrams. We employed ImageJ 1.53v to identify the co-localization of DAPI, SYTOX™ Green and NETs markers, respectively. Six spots from two patients were randomly selected by another researcher to measure the ratio of overlapped area to whole area. A paired samples T-Test was performed using GraphPad Prism version 9.5.0.

## Data Availability

Data generated during the study are within the article or Supplementary Materials.

## Supplementary Materials

The supporting information can be downloaded at: https://www.mdpi.com/article/10.3390/ijms24044101/s1.
