# Supplementary material for: Anti-DNA-IgM Favors the Detection of NET-Associated Extracellular DNA
Source: Int J Mol Sci. 2023 Feb 17;24(4):4101. doi: 10.3390/ijms24044101 (PMC9958910; doi:10.3390/ijms24044101)
Supplement: Supplementary file 1 [file ijms-24-04101-s001.zip › ijms-1993438-supplementary.pdf]

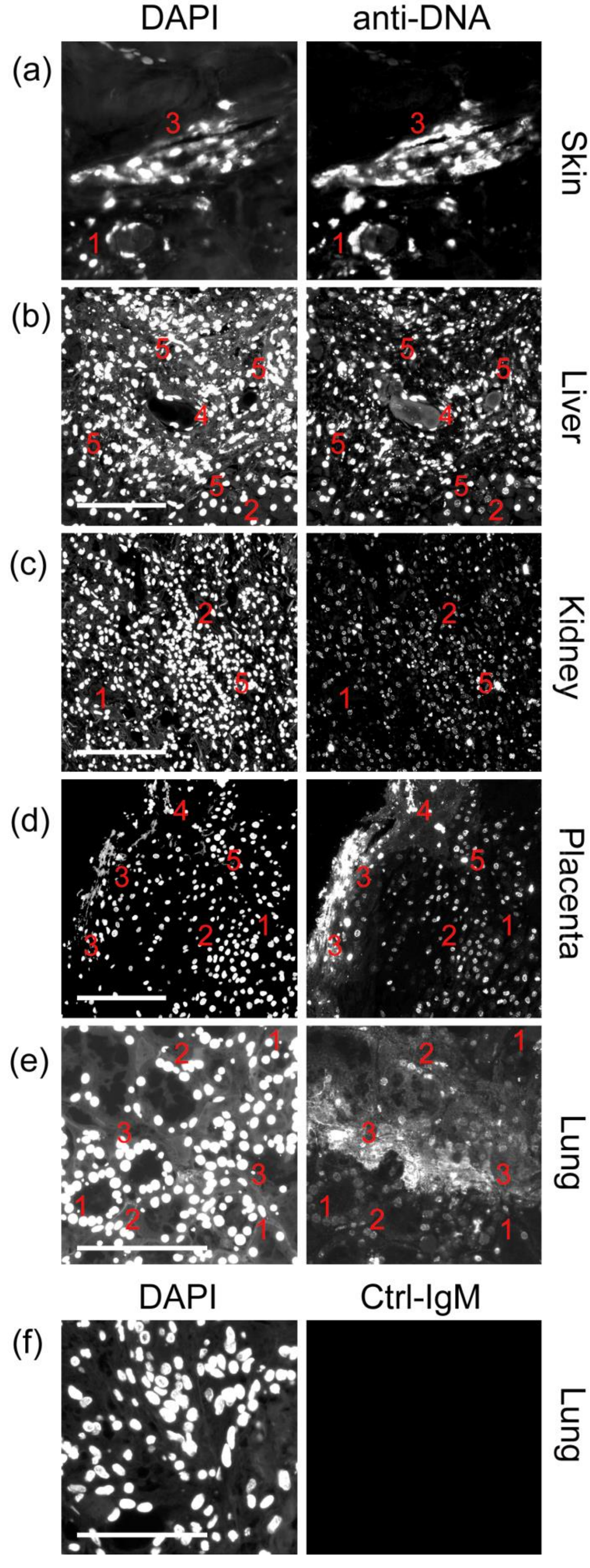

Figure S1. Anti-DNA antibody surpasses DAPI in staining decondensed DNA. We analyzed paraffin sections from specimens of (**a**) skin (n = 7), (**b**) liver (n = 7), (**c**) kidney (n = 7), (**d**) placenta (n = 8),

and (**e**) lung (n = 7) of patients with COVID-19. (**f**) Isotype control. Stains: DAPI (white, left panel), anti-DNA-IgM (white, right panel). Note, condensed nuclei show a strong DAPI signal and (1) no or (2) faint anti-DNA-IgM staining. (3) The spread ecDNA is preferentially stained by the anti-DNA-IgM antibody and (4) some vessels display intravascular DNA which is below the threshold of detection by DAPI. (5) Nuclear remnants in cellular debris are equally stained by DAPI and anti-DNA-IgM. Scale bar = 100 μm.

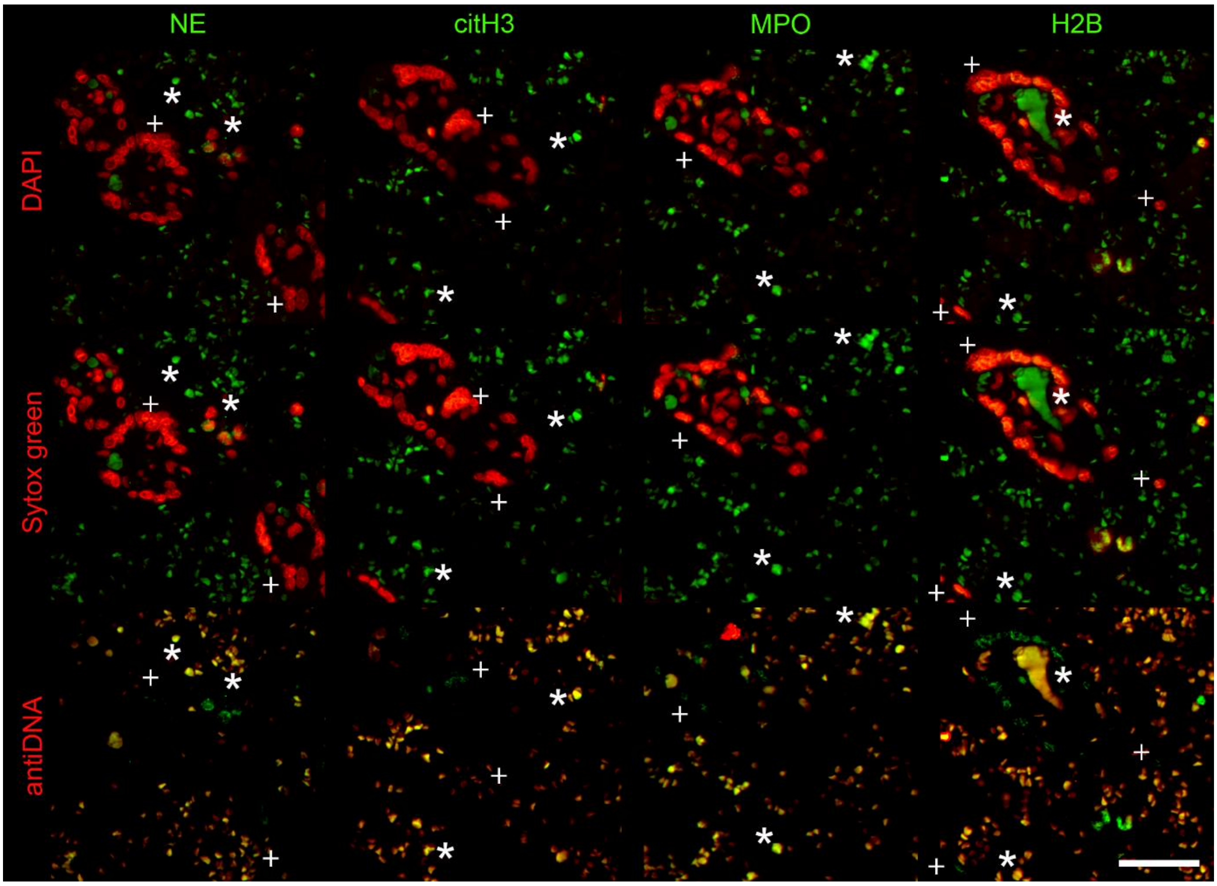

Figure S2. Immunofluorescence staining of NETs in COVID patients. DNA is stained by DAPI (red, first row), SYTOX™ Green (red, second row) and anti-DNA -IgM (red, last row). NET-associated proteins are stained by anti-NE-IgG (green, first column), anti-citH3-IgG (green, second column), anti-MPO-IgG (green, third column) and anti-H2B-IgG (green, last column). NETs are stained by anti-DNA-IgM not DAPI and SYTOX™ Green as seen by the yellow co-localization signal (asterisk). In contrast, anti-DNA-IgM cannot stain nuclei (plus). Scale bar = 50 μm.

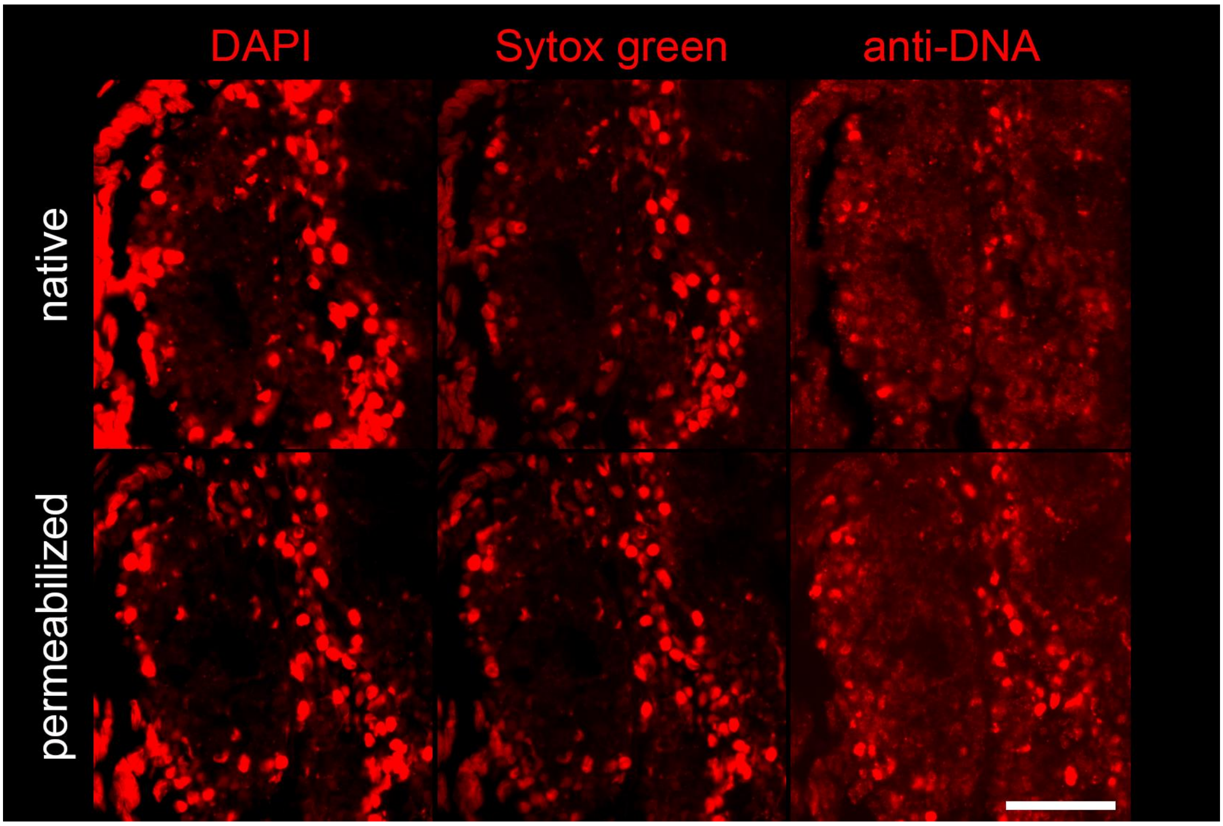

Figure S3. The impact of permeabilization on anti-DNA-IgM antibody immunofluorescence staining of tissues is minimal. Colon carcinoma tissues are stained by DAPI (red), Sytox™ Green (red) and anti-DNA-IgM (red). Scale bar = 100 μm.

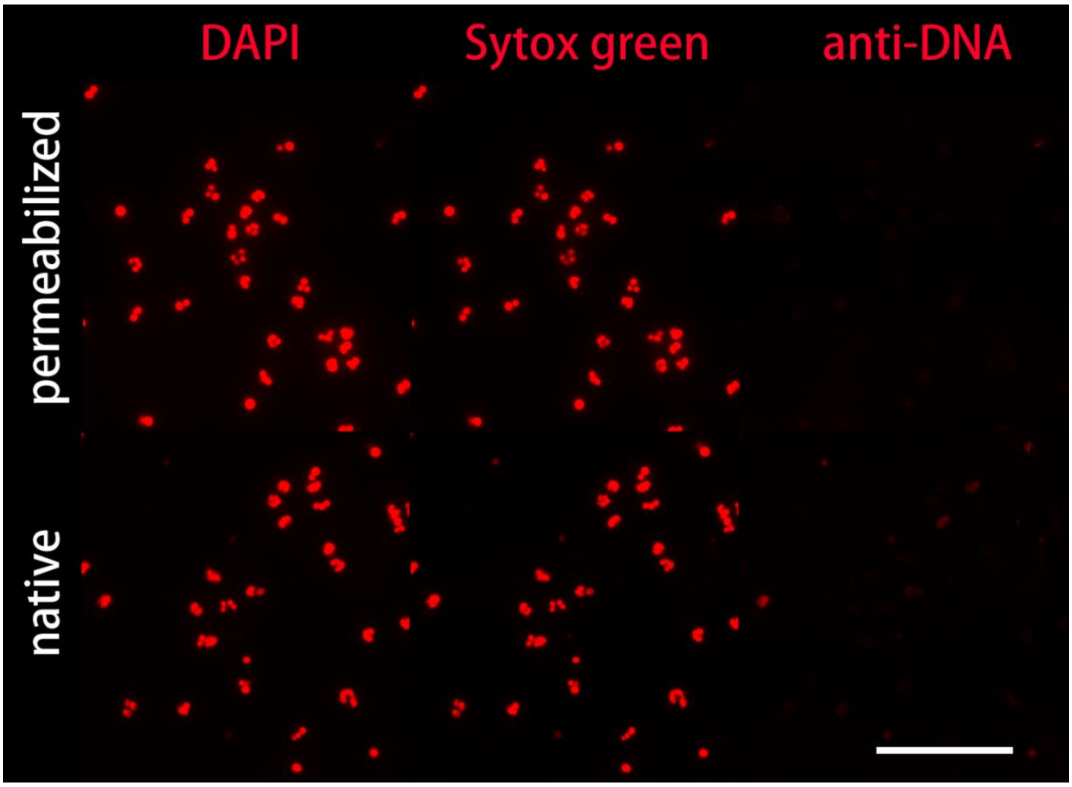

Figure S4. The impact of permeabilization on anti-DNA-IgM antibody immunofluorescence staining of cells is minimal. Unstimulated cells cannot be stained by Anti-DNA-IgM antibody *in vitro*. Stains: DAPI (red), Sytox™ Green (red) and anti-DNA-IgM (red). Scale bar = 100 μm.

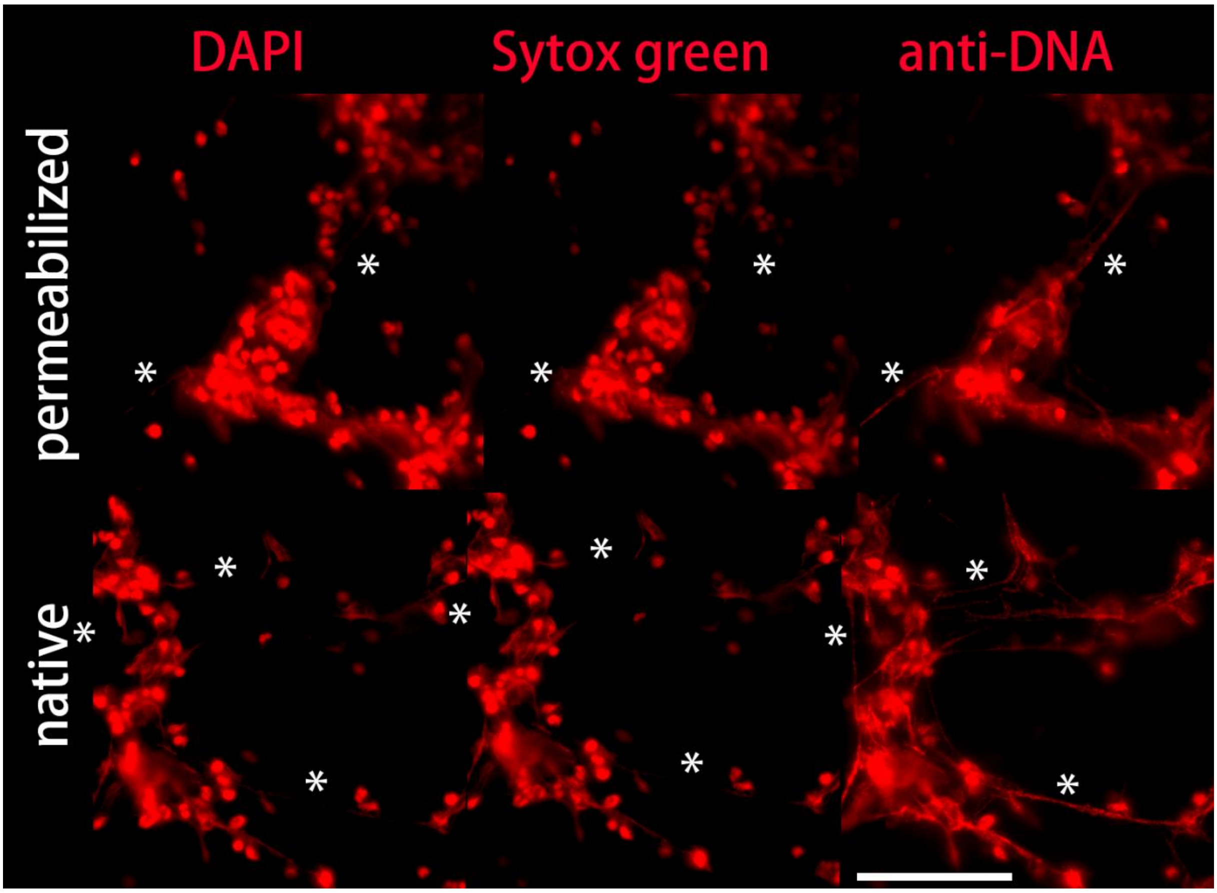

Figure S5. The impact of permeabilization on anti-DNA-IgM antibody immunofluorescence staining of NETs is minimal. NETs are better shown in anti-DNA channel *in vitro* (asterisk). Stains: DAPI (red), Sytox™ Green (red) and anti-DNA-IgM (red). Scale bar = 100 μm.
